# Natural Killer Cells in Hepatic Ischemia-Reperfusion Injury

**DOI:** 10.3389/fimmu.2022.870038

**Published:** 2022-03-28

**Authors:** Miao Huang, Hao Cai, Bing Han, Yuhan Xia, Xiaoni Kong, Jinyang Gu

**Affiliations:** ^1^ Department of Transplantation, Xinhua Hospital Affiliated to Shanghai Jiao Tong University School of Medicine, Shanghai, China; ^2^ Central Laboratory, Department of Liver Diseases, Shuguang Hospital Affiliated to Shanghai University of Traditional Chinese Medicine, Shanghai, China

**Keywords:** NK, natural killer, ischemia-reperfusion injury, liver transplantation, inflammation, immune tolerance

## Abstract

Ischemia-reperfusion injury can be divided into two phases, including insufficient supply of oxygen and nutrients in the first stage and then organ injury caused by immune inflammation after blood flow recovery. Hepatic ischemia-reperfusion is an important cause of liver injury post-surgery, consisting of partial hepatectomy and liver transplantation, and a central driver of graft dysfunction, which greatly leads to complications and mortality after liver transplantation. Natural killer (NK) cells are the lymphocyte population mainly involved in innate immune response in the human liver. In addition to their well-known role in anti-virus and anti-tumor defense, NK cells are also considered to regulate the pathogenesis of liver ischemia-reperfusion injury under the support of more and more evidence recently. The infiltration of NK cells into the liver exacerbates the hepatic ischemia-reperfusion injury, which could be significantly alleviated after depletion of NK cells. Interestingly, NK cells may contribute to both liver graft rejection and tolerance according to their origins. In this article, we discussed the development of liver NK cells, their role in ischemia-reperfusion injury, and strategies of inhibiting NK cell activation in order to provide potential possibilities for translation application in future clinical practice.

## Introduction

Ischemia-reperfusion (I/R) injury is a two-stage pathophysiological process, characterized by hypoxia-induced cell damage in the ischemia phase and by immune inflammation after blood flow restoration (1). The ischemic insult exposes hepatic cells to oxygen deprivation, ATP depletion, and pH changes as well as cellular metabolic stress, all leading to initial cell injury or death (2). During subsequent reperfusion injury, the liver metabolism is disturbed and interconnected inflammatory cascades are induced, thereby further aggravating hepatocellular damage (1). In addition, hepatic I/R can result from both warm and cold ischemia types. Warm I/R occurs in vascular occlusion of liver tissues associated with liver resection, hemorrhagic shock, trauma, cardiac arrest, or hepatic sinusoidal obstruction syndrome (3, 4). However, cold I/R is obviously dominant during liver transplantation, where the donated liver graft is preserved in a hypothermic and anoxic environment before implantation into the recipient (3). Although these two types of liver I/R are involved in different disease models, they share similar characteristics in the mechanism of cell injury, such as activation of liver immune cells after reperfusion (5), among which natural killer (NK) cells play a quite vital role.

NK cells originate from bone marrow, develop in lymphoid tissue, and migrate into the bloodstream and tissues to play a fundamental role in the innate immune response. Discovered in 1975, NK cells are identified to take part in the early defense against virus-infected or tumor cells *via* secretion of granzymes and perforin, or expression of ligands for death receptors without prior immunization (6). In addition, NK cells could also contribute to immunoregulation through producing various cytokines, such as granulocyte-macrophage colony-stimulating factor (GM-CSF), Interleukin (IL) -10, IL-2, C-C motif chemokine ligand (CCL) 3, CCL4, CCL5, Interferon (IFN) -γ, and Tumor Necrosis Factor (TNF) -α (7, 8). It has been previously discussed that NK cells play an important role in other liver diseases, including autoimmune diseases (autoimmune hepatitis, primary biliary cirrhosis, and primary sclerosing cholangitis), non-alcoholic fatty liver disease, and liver fibrosis (9). However, how NK cells affect hepatic I/R is still understood limitedly. Herein, we aim to shed light on the current knowledge in the characteristics of NK cells, their function in hepatic I/R pathogenesis, and the potential mechanism of intervening NK cell activation.

## Development of Hepatic NK Cells

Hepatic NK cells could be mainly divided into circulation NK (c-NK) and liver-resident NK (lr-NK) cell subtypes, which are distinguished by molecular markers, such as (CD27^-^CD11b^+^ and CD27^+^CD11b-) in mouse liver, and CD56^dim^ and CD56^bright^ in human liver (8, 10). In addition, CD49a and CD49b are frequently used to distinguish c-NK (CD49a^−^CD49b^+^Eomes^+^) and lr-NK (CD49a^+^CD49b^−^Eomes^−^) cells in the mouse liver (6). Compared with c-NK, activated lr-NK cells partially retain cytotoxic function to target cells, manifested by reduced expression levels of perforin and granzyme B, but are more efficient in the secretion of TNF-α, IL-2, and GM-CSF (6, 11). In mice, the development of hepatic lr-NK and c-NK cells depends on many different transcription factors, although they are jointly dependent on IL-15 signaling (12). For example, T-bet deficiency only modestly affects peripheral c-NK cell, but has a more severe influence on hepatic lr-NK cells (12, 13). Mice lacking *Nfil3* have a quantitatively significant decrease in the bone marrow NK progenitor and mature NK cells, however, *Nfil3* is dispensable for lr-NK (14–17). Moreover, in contrast to *Eomes* deficiency reducing bone marrow and peripheral c-NK cell numbers, *Eomes* are not required for hepatic lr-NK development (18, 19). Promyelocytic leukemia zinc finger (PLZF) and aryl hydrocarbon receptor (AhR) have been reported to promote the development of different lr-NK cells, while these two transcription factors are not critical for c-NK development (20, 21). In other words, mice lacking PLZF or AhR have reduced hepatic lr-NK numbers, but c-NK are not significantly altered.

Some murine NK cell markers do not correspond one-to-one with their human counterparts, which largely explains the lack of phenotypic matching between murine and human lr-NK cells. Overall, c-NK cells rely mainly on T-bet and Eomes for humans, which has some similarities to the developmental process of mice, whereas lr-NK cells are regulated by a wide range of transcription factors including Eomes, Hobit, etc (11, 22). Compared with mice, human lr-NK cells express high levels of Eomes but not T-bet (11, 23, 24). Specifically, human lr-NK cells have few positive expressions of T-bet and are all positive in Hobit (25, 26). It is worth noting that lr-NK cells are probably dependent on the expression of surface adhesion molecules such as CD69, CD103, CD49a, CCR5, CXCR3, and CXCR6 to support their residence in the liver sinusoids (27, 28).

## Function of NK Cells in Hepatic I/R

**Figure 1:**
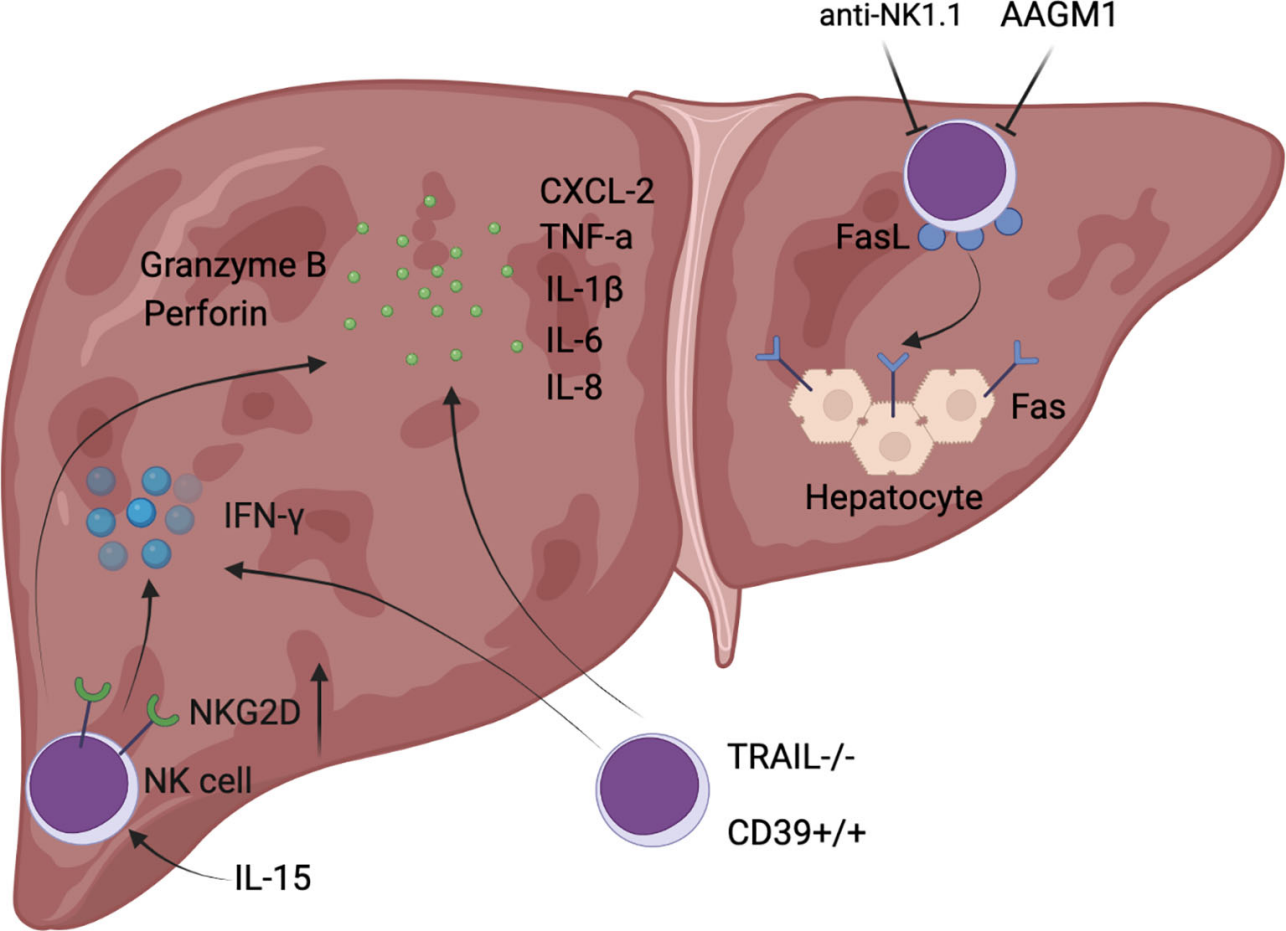
Diagrammatic sketch of NK cell-mediated exacerbated liver injury during hepatic I/R.

**Figure 2:**
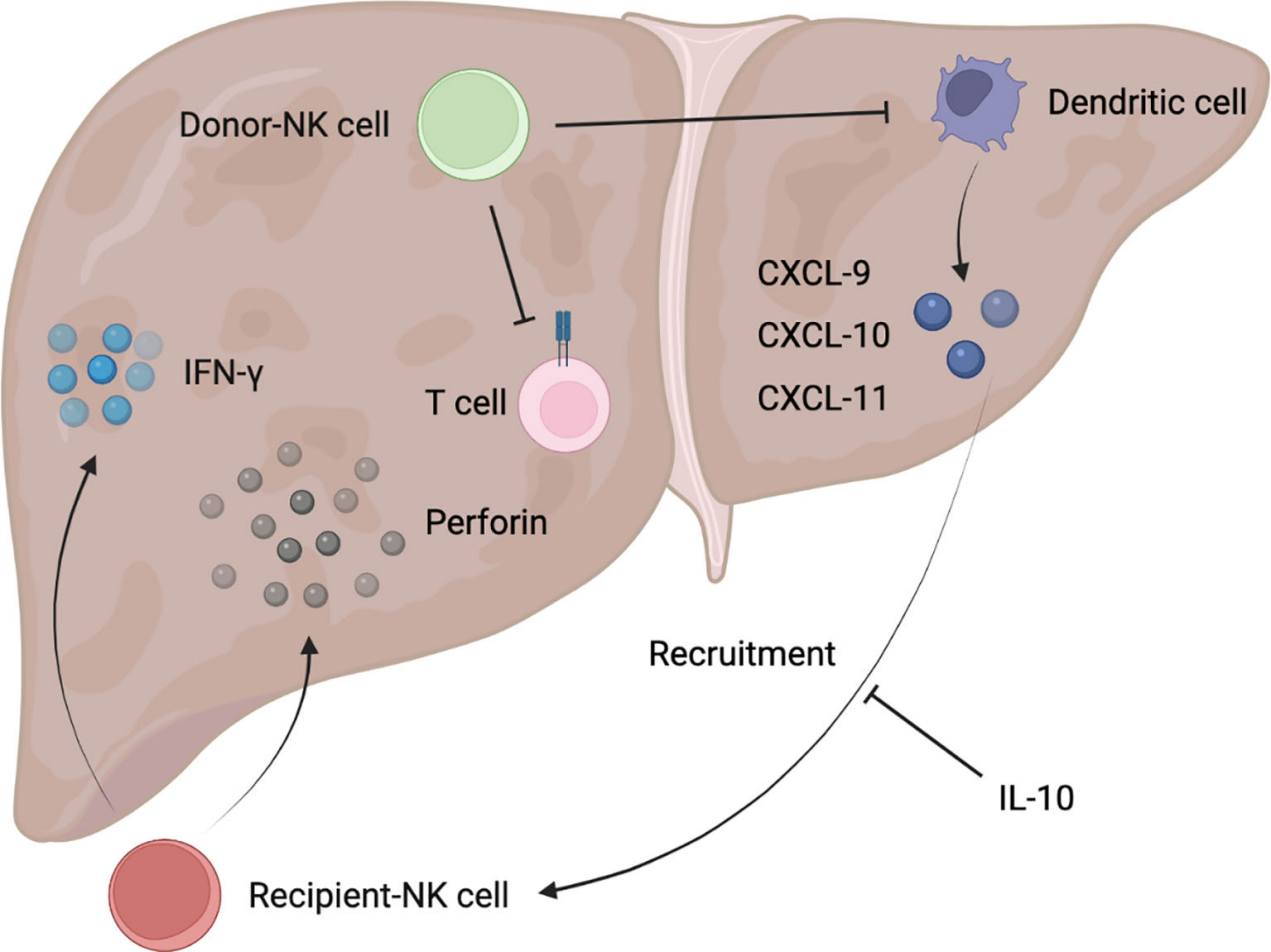
Schematic diagram of donor/recipient-derived NK cells in immune tolerance after liver transplantation.

NK cell signaling in hepatic I/R is primarily mediated by engagement of their activating receptors (mainly including CD16, NKG2C:CD94, NKG2D, NKp46, NKp44, NKp30, CD226, and natural killer granule 7) and inhibitory receptors (largely composed of CD96, TIGIT, LAG3, killer cell immunoglobulin receptor (KIR) (Ly49 receptor in mice), and NKG2A:CD94), and the dynamic balance between them determines the responsiveness of NK cells (29, 30). Notably, the human KIR (Ly49 molecule in mice) and NKG2A:CD94 could recognize MHC class I molecules that are abundantly expressed on normal cells, thereby inhibiting NK cell function to ensure perfect tolerance to their own healthy cells (29, 31). In warm I/R, such as liver resection and hemorrhagic shock, NK cells mainly play the role of aggravating liver injury and promoting inflammatory cell infiltration (**Figure 1**). Whereas in cold I/R (primarily liver transplantation), NK cells not only participate in the inflammatory response, but also produce a marked effect in post-transplant immune tolerance (**Figure 2**).

### NK Cells and Inflammatory Response in Hepatic I/R

It has been previously reported that depletion of NK cells had no significant effect on hepatic I/R injury, as demonstrated by unchanged liver function assays and infiltrated MPO content into the liver, suggesting that NK cells are not recruited to the liver after reperfusion (32). However, there is accumulating direct evidence that NK cells are involved in the development of the pathophysiology of hepatic I/R. In an experiment in which a rat was subjected to 1 hour of right hepatic lobe ischemia and the remaining liver was excised, a significant increase in NK cell infiltration into the rat liver parenchyma was detected after reperfusion for 2 hours (33), and depletion of NK cells could significantly decrease hepatic CXCL-2 expression, reduce neutrophil infiltration into the liver, and attenuate hepatic I/R injury levels (34). Similarly, the mRNA level of *NKG2D*, an activating receptor for NK cells, rapidly increased in allografts (LEW to SD rats), indicating a significant activation of NK cells, while donor livers that were pretreated with anti-asialo monosialotetrahexosylganglioside (AAGM1) to deplete NK cells, showed a significant decrease in not only NKG2D expression levels but also hepatic cytokines (TNF-α, IL-1 β, IL-6, and IL-8) as well as secretion levels of cytolytic molecules (perforin, granzyme B), along with a reduction in early neutrophil infiltration (31). In contrast to the depletion of NK cells described above, administration of soluble pro-inflammatory IL-15/IL-15Rα complexes increased the absolute number of NK cells in the liver of *IRF-1* knockout mice after orthotopic liver transplantation, accompanied by increased expression of cytotoxic effector molecules (NKG2D, granzyme B, and perforin) and inflammatory cytokines (IFN-γ, IL-6, and TNF-α), eventually resulting in worsening liver injury and decreased survival (35). NK cell activation in hepatic I/R relied on the hydrolysis of ADP to AMP by CD39, and *CD39* deletion reduces IFN-γ secreted by NK cells to limit hepatic I/R injury (5). To be specific, *CD39*-deficient mice exhibited reduced pro-inflammatory cytokines in serum 3 hours after reperfusion, and also showed reduced hepatocyte injury and necrosis area after 24 hours of reperfusion (5). In addition, TRAIL expression on NK cells was upregulated following hepatic I/R in mice and exerted marked protective effects such as reduced serum transaminases, histological necrosis, neutrophil infiltration, and IL-6 levels in serum, whereas TRAIL-null NK cells exhibited higher cytotoxicity and significantly increased secretion of IFN-γ, indicating that TRAIL could confine further liver injury by blocking NK cell activation (36). IFN-γ secreted by NK cells can upregulate the expression of Fas in hepatocytes, while IL-18 released by injured Kupffer cells in hepatic I/R injury could increase the expression level of FasL (Fas ligand) in NK cells (37, 38). Therefore, the inflammation and liver damage are further amplified under this complex positive feedback. As can be seen from the above literature, IFN-γ secretion by NK cells plays a pivotal role in hepatic I/R, and the mechanism of its secretion is largely regulated by Tbox transcription factors such as T-bet and Eomes (39), which is tightly associated with NK cell development. NK cells can not only aggravate hepatic I/R injury by producing IFN-γ, but also increase the synthesis of IL-17, which could enhance the recruitment of neutrophils into the injured liver (34). This study provided strong proof that neutralization of IL-17 attenuated hepatic I/R injury in *Rag1* knockout mice (34).

### NK Cells and Immune Tolerance in Hepatic I/R

The immunological process of donor NK cells entering the recipient bloodstream and recipient NK cells flowing into the donor graft with the bloodstream occurs early after liver transplantation, where donor NK cells migrate out of the liver and are detected in the recipient’s circulation generally for 2 weeks, but there are also possibilities that graft NK cells will persist for decades (40). Thirteen genes were found to be significantly overexpressed in NK cells from immune-tolerant recipients of liver transplantation, suggesting that NK cells may be involved in the induction of immune tolerance (41). In fact, recipient-derived NK cells and donor-derived NK cells could play opposed roles in liver graft tolerance, with the former tending to reject allografts and the latter mainly promoting tolerance (40, 42). Recipient-derived NK cells produced IFN-γ after entering liver grafts, which predisposed to graft non-function (43). Correspondingly, either depletion of NK cells or reduction of IFN-γ production could be capable of promoting increased graft survival rate (43). According to clinical trials, recipient hypertension can further activate NK cells and lead to moderate to severe I/R injury, which may markedly increase the incidence of early allograft dysfunction and reduce the 6-month survival rate of grafts (44, 45). In contrast, after liver transplantation, treatment with anti-inflammatory factor IL-10 given to the recipient reduced the NK recruitment of chemokines CXCL-9, CXCL-10, and CXCL-11 produced by activated Dendritic cells, leading to a decrease in the number of recipient NK cells entering the liver graft in a clinical study (46). Meanwhile, a decrease of pro-inflammatory IL-12 also induced a shift in recipient NK cells to a tolerogenic phenotype with concomitant downregulation of NK-activating receptors, and reduction of cytotoxicity and cytokine production (47), which further limits the rejection of transplanted liver mediated by recipient NK cells. Of note, the rejection of ABO-incompatible liver transplantation by NK cells was particularly pronounced in clinical practice. It has been reported that a large number of NK cells in recipient peripheral blood was the only risk factor for the induction of ischemic biliary tract disease after ABO-incompatible adult living donor liver transplantation. This may be explained that recipient NK cells could recognize various NK cell ligands on the donor endothelial cells after flowing into the liver graft, and the ABO antigen was abundantly expressed on the endothelial cells of the transplant graft, thereby directly producing cytotoxicity and resulting in decreased graft survival (48, 49). Besides, there is a clinical study suggesting that recipient-derived NK cells are reduced but retain the robust expression of NK cell receptors such as NKG2D in the early stage of pediatric liver transplantation, which may be closely related to the increase of acute graft rejection episode (50).

As previously reported, infusion of donor liver NK cells can alleviate acute rejection of rat liver allografts and prolong graft survival (51), revealing the important function of donor NK cells in immune tolerance. It has also been reported in clinical research that bone marrow-derived mesenchymal stromal cell infusion induces the increase of donor-derived NK cells, which promotes the establishment of a pro-tolerance graft environment that persists in a long time (52). Hepatic NK cells from the donors played a major role in promoting graft tolerance, possibly through direct killing of recipient activated T cells and immature Dendritic cells recruited to the transplanted liver (40, 53). But this requires more experimental evidence to further confirm.

## Strategies to Inhibit NK Cells in Hepatic I/R

On the one hand, NK cells play a pro-inflammatory and pro-injury role during hepatic I/R. On the other hand, donor NK cells will be gradually replaced by recipient NK cells after liver transplantation, which predominantly exerts the negative effect of mediating graft liver rejection. Therefore, it is necessary to intervene in NK cells during the occurrence of I/R in the liver and at the stage of immune rejection that follows. Currently, possible approaches are NK cell depletion, inhibition of NK cell activation receptor signaling, and blockade of NK cell developmental signaling.

### NK Cell Depletion

In general, there were two commonly used depletion antibodies for NK cells, AAGM1 and anti-NK1.1 (34, 54). AAGM1 causes certain membrane damage that gives rise to loss of NK function, and is effective in depleting NK cells in various mouse strains, but it may also interfere with other lymphocyte subsets that express GM1 (54). Treatment with the anti-NK1.1 antibody PK136 depletes NK cells in the liver and in a subset of NKT and γδ T cells (34). However, anti-NK1.1-mediated NK cell depletion is still the mainstay of analysis of NK cells in the liver (34). In a phase I/II trial, NK cells are depleted by a combination of anti-CD3 and anti-CD7 antibodies to treat acute graft-versus-host disease (55).

### NK Cell Activating Receptor Blockade

It has been reported that transforming growth factor-β, IL-10, tryptophan catabolites, prostaglandin E_2_, dickkopf-related protein 2, indoleamine 2,3-dioxygenase, soluble HLA-G, soluble NKG2D ligands, and galactin-3 (soluble inhibitory receptor for NKp30) could be viewed as the inhibitors of NK cells and their receptors, downregulating cytotoxic activity and the capabilities of secreting IFN-γ (56). For example, corticosteroids are utilized to significantly downregulate the expression of activated receptors NKp30 and NKp46 in clinical research, and NKG2D blockade can also reduce the incidence of allograft rejection (55). However, there are still many aspects of validation necessary to apply the above methods to the clinic.

### Blockade of NK Cell Developmental Signaling

It has been reported that multiple signaling pathways are involved in the activation of NK cells and mediate the important role of NK cells in liver I/R. For example, activated AMPK, deletion of *FoxO1* gene, and inhibition of SREBP all could downregulate the number of mature terminally differentiated NK cells, inhibit NK cell cytotoxicity, and reduce granzyme B and IFN-γ production expression levels (57, 58). But these pathways are far from well-studied and full of controversy and contradictions. At present, the mTOR pathways have been widely and relatively well studied, and the metabolic signaling mediated by it is generally considered to be an important node in NK cell development. mTOR is a ubiquitous serine/threonine kinase that requires two regulatory proteins, raptor and rictor, to form functionally distinct mTOR complexes 1 and 2 (mTORC1 and 2), respectively (59). mTORC1 plays a major role among mTOR pathways, whereas mTORC2 can negatively regulate mTORC1 activity to some extent by inhibiting STAT5-mediated expression of the amino acid transporter SLC7A5 (60, 61). IL-15 stimulated mTORC1 through the Jak1 and PI3K/Akt signaling axes, and activated mTORC1 promoted NK precursor development (62, 63). Moreover, mTOR activated by the kinase PDK1, downstream of IL-15 signaling, was found to be required for E4BP4 expression in bone marrow NK cells, and E4BP4 can promote *Eomes* transcription, thereby playing an indispensable role in NK cell development (15). Since mTOR activity was inhibited after knockdown of *PDK1* in NK cells, NK development was impeded at an early stage (64). When *mTOR* was specifically knocked out in NK cells, the number of NK cells in peripheral blood was drastically reduced (65). Moreover, the use of the mTOR-selective inhibitor rapamycin broadly inhibited the expression levels of IFN-γ, perforin, and granzyme B secreted by NK cells (66, 67). In a rat I/R study, rapamycin significantly reduced parenchymal infiltration of NK cells, liver histological damage, and mortality (33), which formed strong support for the above observations.

## Conclusions and Future Perspectives

In clinical practice, an important factor affecting the prognosis of partial hepatectomy and liver transplantation is liver I/R. The former is mainly manifested as severe liver injury caused by I/R, while the latter is also associated with graft rejection. Considering the inevitability of I/R in this type of liver surgery, mitigating the hazards caused by I/R to patients becomes the key. Accumulating evidence indicates that NK cells can be recruited to the liver, activate inflammation and worsen liver injury in I/R, and enhance rejection of grafts by recipient NK cells, thereby reducing graft survival. Using NK cell depletion, inhibiting NK cell activating receptors, or blocking the signaling pathway of NK cell maturation will become an effective approach for the intervention of hepatic I/R, which may show great potential for the clinical application. Collectively, an understanding of the pro-inflammatory effects of NK cells, as well as the donor/recipient-derived immune tolerance/rejection, may aid in liver protection in liver transplantation and partial hepatectomy, providing a rationale for further clinical treatments in the future.

## Author Contributions

JG and XK conceived the topic of this review article. MH, HC, BH, and YX searched reference articles and extracted key information for this review article. MH and XK wrote this manuscript. All authors listed have made a substantial, direct, and intellectual contribution to the work, and approved it for publication.

## Funding

This work was supported by the National Natural Science Foundation of China (82130020, 82072646, and 81772507 to JG, 81873582 and 82070633 to X Kong). Clinical Research Plan of SHDC (No. SHDC2020CR3005A), Shanghai “Rising Stars of Medical Talent” Youth Development Program “Outstanding Youth Medical Talents” (No. SHWSRS (2021) _099), Shanghai Municipal Education Commission–Gaofeng Clinical Medicine Grant Support (No. 20191910) to JG. Program of Shanghai Academic/Technology Research Leader (20XD1403700) to XK.

## Conflict of Interest

The authors declare that the research was conducted in the absence of any commercial or financial relationships that could be construed as a potential conflict of interest.

## Publisher’s Note

All claims expressed in this article are solely those of the authors and do not necessarily represent those of their affiliated organizations, or those of the publisher, the editors and the reviewers. Any product that may be evaluated in this article, or claim that may be made by its manufacturer, is not guaranteed or endorsed by the publisher.

## Abbreviations

AAGM1, anti-asialo monosialotetrahexosylganglioside; AhR, aryl hydrocarbon receptor; CCL, C-C motif chemokine ligand; GM-CSF, granulocyte-macrophage colony-stimulating factor; IFN, interferon; IL, Interleukin; I/R, ischemia-reperfusion; KIR, killer cell immunoglobulin receptor; NK, natural killer; PLZF, promyelocytic leukemia zinc finger; TNF, tumor necrosis factor.
